# Assessment of Air Polishing with Erythritol Powder on the Removal of Biofilm and Dental Calculus: An Ex Vivo Pilot Study

**DOI:** 10.3390/dj14100643

**Published:** 2026-10-05

**Authors:** Charles M. Cobb, Stephen K. Harrel, Donggao Zhao

**Affiliations:** 1 Department of Periodontics, School of Dentistry, University of Missouri-Kansas City, 650 E. 25th Street, Kansas City, MO 64108, USA; 2 Department of Periodontics, School of Dentistry, Texas A&M University, 3302 Gaston Avenue, Dallas, TX 75246, USA; skharrel@gmail.com; 3 Electron Microscope Core Facility, School of Dentistry, University of Missouri-Kansas City, 650 E. 25th Street, Kansas City, MO 64108, USA; zhaodo@umkc.edu

**Keywords:** periodontal diseases, periodontitis, biofilms, dental calculus, erythritol, scanning electron microscopy

## Abstract

**Background:** Air polishing with erythritol powder has recently been promoted as a method for removing calculus and biofilm in periodontal pockets of up to 9 mm depth. This ex vivo study was conducted to determine the impact of erythritol air polishing, for various time periods, on subgingival calculus and associated biofilm. **Methods:** Specimens consisted of 33 extracted teeth. Test sites with visible calculus were circumscribed using a 4 mm trephine bur on the root surface of each specimen. Positive controls were treated with air–water spray only. Test site treatment consisted of air polishing with erythritol powder with a 14 µm particle size. Fifteen positive control and 15 test specimens were subdivided into 3 groups of 5 specimens each and treated for 5, 10 and 15 s, respectively. The remaining three specimens were untreated negative controls. All specimens were examined using SEM. Selected specimens were subjected to EDS analysis. **Results:** Calculus was observed in all specimens across all treatment groups. Bacteria embedded in calculus and the extracellular polymeric matrix were consistently present across treatment groups. Treatment duration was associated with a reduction in surface biofilm, but no duration of treatment eliminated calculus or deeply embedded bacteria. **Conclusions:** The results of this ex vivo pilot study suggest that erythritol air polishing of calculus-laden root surfaces does not completely remove subgingival calculus or calculus-embedded microbes. Following treatment, smear layers with embedded microbes were noted on 55% of specimens. The study results suggest that erythritol air polishing protocols, in a clinical setting, may still be dependent on subgingival scaling and root planning to achieve the traditional endpoints of periodontal therapy.

## 1. Introduction

In 2017, a commercially sponsored evidence-based consensus report addressed the safety and efficacy of using an air–powder polishing device for supra- and subgingival applications during dental prophylaxis and periodontal maintenance [1]. The report did not directly address the removal of dental calculus. Instead, the consensus report stated, “it is generally understood that air polishing is not an effective or efficient method for the removal of dental calculus [1].” Further, it was noted that “air polishing should be used on calculus-free surfaces or after existing calculus has been removed with the use of more aggressive types of instrumentation [1,2,3,4].” However, air polishing with erythritol powder has recently been promoted as removing supra- and subgingival “young” calculus and biofilm in subgingival periodontal pockets of up to 9 mm depth [5].

The concept of “young” calculus is seldom encountered in the dental literature and remains ill defined. Based on the relative age of calculus deposits, terms such as young, immature, mature and old have been utilized. Schroeder and Bambauer [6], in their study on the stages of calcium phosphate crystallization during calculus formation, collected samples of supragingival “young” calculus with an age of between 3 and 300 days and “old” calculus with an age ≥ 365 days. Tan et al. [7] defined young calculus as having formed within 12 weeks following a professional prophylaxis. Mature calculus was defined as a subgingival deposit in subjects with periodontal disease who had not received treatment in six or more months prior to sample procurement.

Regardless of the age of calculus, biofilm composition appears to be more dependent on location. The biofilms associated with supra- and subgingival calculus can be significantly different [8,9,10]. For example, supra- and subgingival biofilms exhibit differences in composition, the former being more aerobic and comprising a variety of Gram-positive *Streptococci* and *Actinomyces* species. In contrast, subgingival biofilm is dominated by anaerobic, Gram-negative microbes. In the case of periodontitis, with increasing attachment loss, the orange and red complex microbes assume more dominance, e.g., *Porphyromonas gingivalis*, *Treponema denticola*, *Tannerella forsythia*, *Filifactor alocis*, *Parviomonas micra*, *Fusobacterium* spp., *Prevotella* spp., *Campylobacter* spp., and *Eubacterium* spp. [11].

Thus, it appears that newly formed, immature calculus is supragingival, softer, less dense, and exhibits a different mineral content and Ca/P ratio and a different composition of the attendant biofilm than does older, mature subgingival calculus [6,7,12,13].

Given the differences in location (supra- vs. subgingival), structural features, and chemical composition of immature vs. mature calculus and the inference that air polishing can be used for the removal of both supra- and subgingival calculus and attendant biofilms, this ex vivo study was conducted to determine the impact of air polishing with erythritol powder, for various time periods, on subgingival calculus and the associated biofilm.

## 2. Specimens and Methods

### 2.1. Specimen Preparation

Specimens consisted of 33 teeth extracted due to severe periodontal disease. No patient identifiers were associated with the teeth. The study protocol was submitted to the Institutional Review Boards (IRB) of the Texas A&M University Health Science Center, Dallas, Texas, USA. and the University of Missouri—Kansas City, Kansas City, Missouri, USA. It was determined by both institutions that the study was not considered human subject research and that informed consent or IRB approval was not necessary.

Specimens met the following inclusion criteria: (1) no root caries; (2) presence of visible calculus; (3) no visible root fractures or anatomical abnormalities; and (4) a root surface that was relatively flat and presented an adequate area for treatment. Immediately upon extraction, specimens were rinsed free of blood using cold 0.9% saline, assigned a random code number, and stored until treatment in individual bottles of 10% neutral buffered formalin. Prior to treatment, all specimens were rinsed again with sterile water.

### 2.2. Test Site

Test sites were prepared as described in a previous study [14]. Using a 4 mm trephine bur, a single test site, covered with visible calculus, was prepared on the selected root surface of each specimen. Under copious irrigation with sterile water, the trephine was used at slow speed to cut a circular trough through the calculus and into the root surface, creating a uniform test area measuring 3.9 mm in diameter with a surface area of ∼11.95 mm^2^. Following preparation of the test area, the specimen was rinsed twice with sterile water using an irrigation syringe. Loose debris was removed using compressed air.

### 2.3. Treatment

An air polishing unit (EMS AirFlow^®^ Handy 3.0 Handheld Air Polish Device, EMS Electro Medical Systems S.A., Nyon, Switzerland) with a nozzle (EMS PERIOFLOW^®^ Nozzle, EMS Electro Medical Systems S.A., Nyon, Switzerland) designed for subgingival applications was used for all positive control and test sites. According to the manufacturer, water and air pressure ranges from 2.7 to 3.5 bar (~39.2 to ~50.7 PSI as 1 bar ≈ 14.5 PSI) were utilized [15]. The angle of application of the subgingival tip in a clinical situation is dictated by the soft tissue wall of the periodontal pocket, the orientation being roughly parallel to the tooth root. In the ex vivo model, the subgingival tip (EMS) was placed next to the test area, mimicking the manufacturers’ recommendation for use of the tip subgingivally, i.e., parallel to the specimen surface [16]. The tip was then moved over the test area as recommended by the manufacturer. This action closely followed what would occur during the use of the tip to access a periodontal pocket.

**Figure 1:**
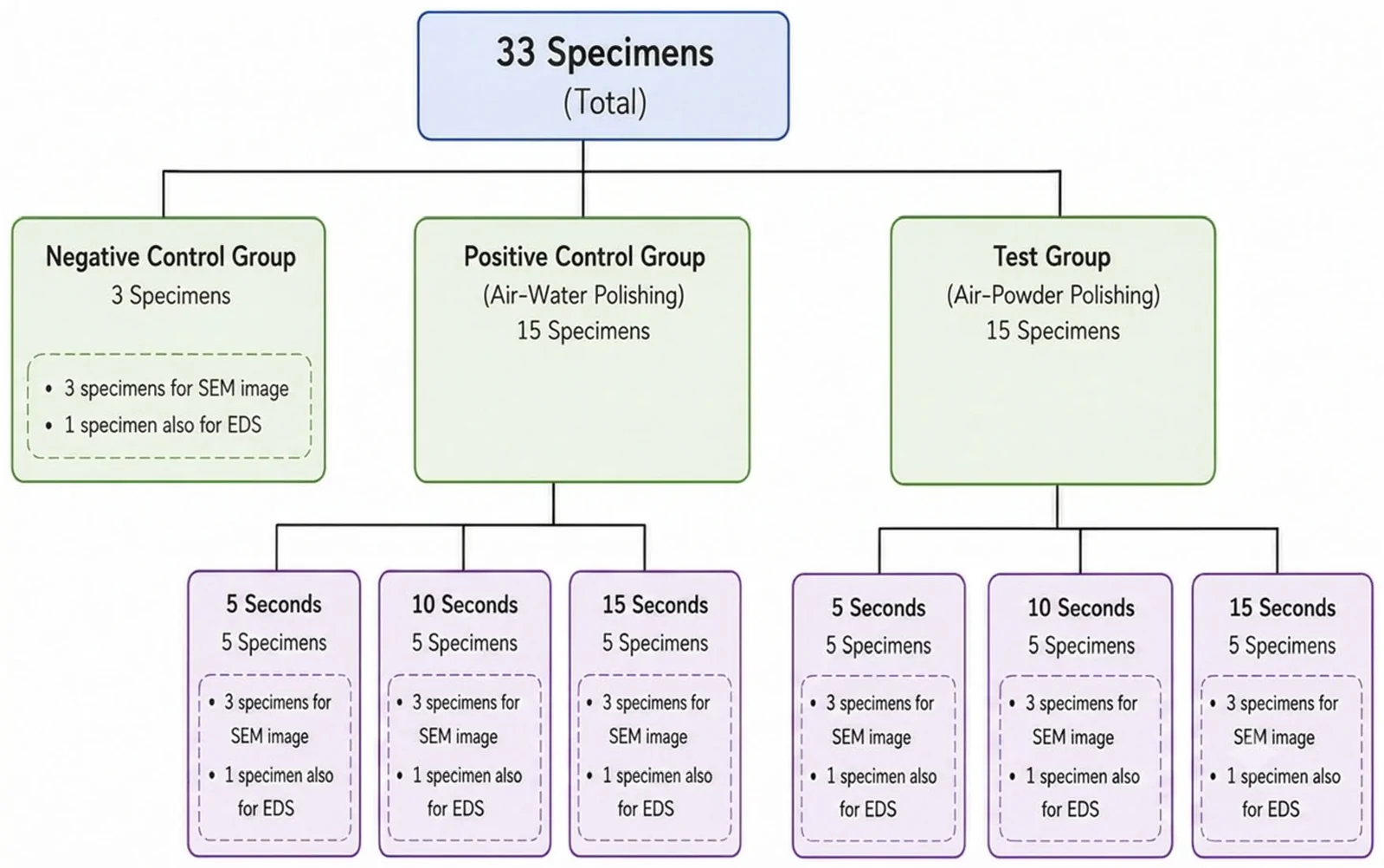
Diagram showing allocation and number of specimens examined by SEM and EDS.

For the positive control specimens, treatment consisted of air polishing spray using water only. Test site treatment consisted of air polishing spray with water and erythritol powder with a 14 µm particle size (EMS Air Flow Perio Powder, EMS Electro Medical Systems S.A., Nyon, Switzerland). Fifteen positive control and 15 test specimens were subdivided into 3 groups of 5 specimens each and treated for 5, 10 and 15 s, respectively. The remaining 3 specimens were untreated negative controls (Figure 1).

### 2.4. Scanning Electron Microscopy (SEM)

Three negative control specimens, 9 positive control specimens and 3 specimens from each of the three timed test treatment groups were randomly selected for SEM imaging, a total of 21 specimens (Figure 1). Selected specimens maintained their code number, enabling masking of the examiners responsible for the evaluation of imaging results. Further, the code numbers were used for the random selection of specimens for SEM examination. Specimen preparation followed a routine protocol for biological specimens, as detailed previously by Cobb et al. [17]. The protocol essentially consists of (1) a thorough rinse in sterile water; (2) dehydration in a series of graded ethyl alcohol solutions (33–100%); (3) specimens mounted on aluminum stubs and stored in a desiccator until examined by SEM; and (4) specimens being sputter-coated with approximately 200 Å of gold–palladium immediately prior to SEM examination. Images were acquired using a Philips XL30 ESEM-FEG SEM (FEI Corp., North America NanoPort, Hillsboro, OR, USA) located at the University of Missouri—Kansas City, USA. The instrument was upgraded with a Windows II-based SEMView8000 software system (SEMTech Solutions Inc., North Billerica, MA, USA). SEM parameters were as follows: an accelerating voltage of 10 kV, working distance of approximately 10 mm, and a spot size setting of 3. The trephine-inscribed root surface was examined at 50× magnification for orientation, to identify areas of interest and assess overall surface structure. Areas of interest, surface characteristics and residual calculus and biofilm were photographed at magnifications of 50×, 200×, 500×, 1000× and 5000×. An aggregate total root surface area of ∼18.4 mm^2^ was evaluated for each of the three specimens in each control and each treatment group.

### 2.5. Energy Dispersive Spectrometry (EDS)

The XL30 SEM was equipped with an EDAX Octane Elect Super detector and APEX Advanced software (METEK Materials Analysis Division, AMETEK, Inc., Mahwah, NJ, USA) which allow for the determination of the elemental composition of one specimen each from the test groups, i.e., 5, 10 and 15 s air polished with erythritol; one specimen from the untreated negative control group; and one sample of unused erythritol powder. The semiquantitative EDS data of weight % and atomic % was obtained from four separate areas on each sample and averaged for the purpose of comparison.

Parameters for the EDS analysis were an accelerating voltage of 15 kV, a working distance of approximately 10 mm, a 150 nm beam size, a takeoff angle of 35°, and a magnification of 50×. Counts per second (CPS) ranged from approximately 5000 CPS for the erythritol powder specimen to 62,000 CPS for the negative control specimens, to 74,000 CPS for the 5, 10 and 15 s test group specimens. Elements of interest were calcium (Ca), phosphorus (P), carbon (C), oxygen (O), sodium (Na), magnesium (Mg), and silicon (Si).

### 2.6. Analysis of Data

Given the small sample base and the pilot study nature of the project, formal statistical analysis of data was not indicated and a power analysis was not performed. Thus, the analysis of SEM and EDS data was qualitative and not quantitative. Two investigators (CMC and DZ), with a combined 96 years of experience in SEM and EDS instrumentation, carried out the analysis. Neither investigator was formally calibrated regarding interpretation of the SEM data.

## 3. Results

One specimen in the 5 s positive control group was damaged during processing. Consequently, in this group, the three specimens for SEM examination were selected from the remaining four. The investigator responsible for the SEM examination (CMC), prior to breaking the code, assigned each specimen to a treatment group, based on the results of the SEM assessment. A concurrence rate, i.e., correct assignment, of only 15% (3 of 20) was achieved.

After breaking the code, it was determined that the discarded specimen belonged to the 15 s positive control group.

### 3.1. SEM Image Analysis

Specimens were evaluated for surface topography, texture, and biofilm. Specifically, surface characteristics included the following: (1) presence of calculus; (2) presence of a smear layer; (3) presence of instrumentation marks; (4) presence of a granular debris field; (5) smooth, flat, rounded or rough surface; (6) porous surface texture; (7) presence of biofilm; (8) bacteria embedded in calculus; and (9) presence of extracellular polymeric matrix (ECPM). If biofilm was present, morphotypes of bacteria were identified, e.g., cocci; short, medium, or long rods; fusiform; and spirochetal.

**Figure 2:**
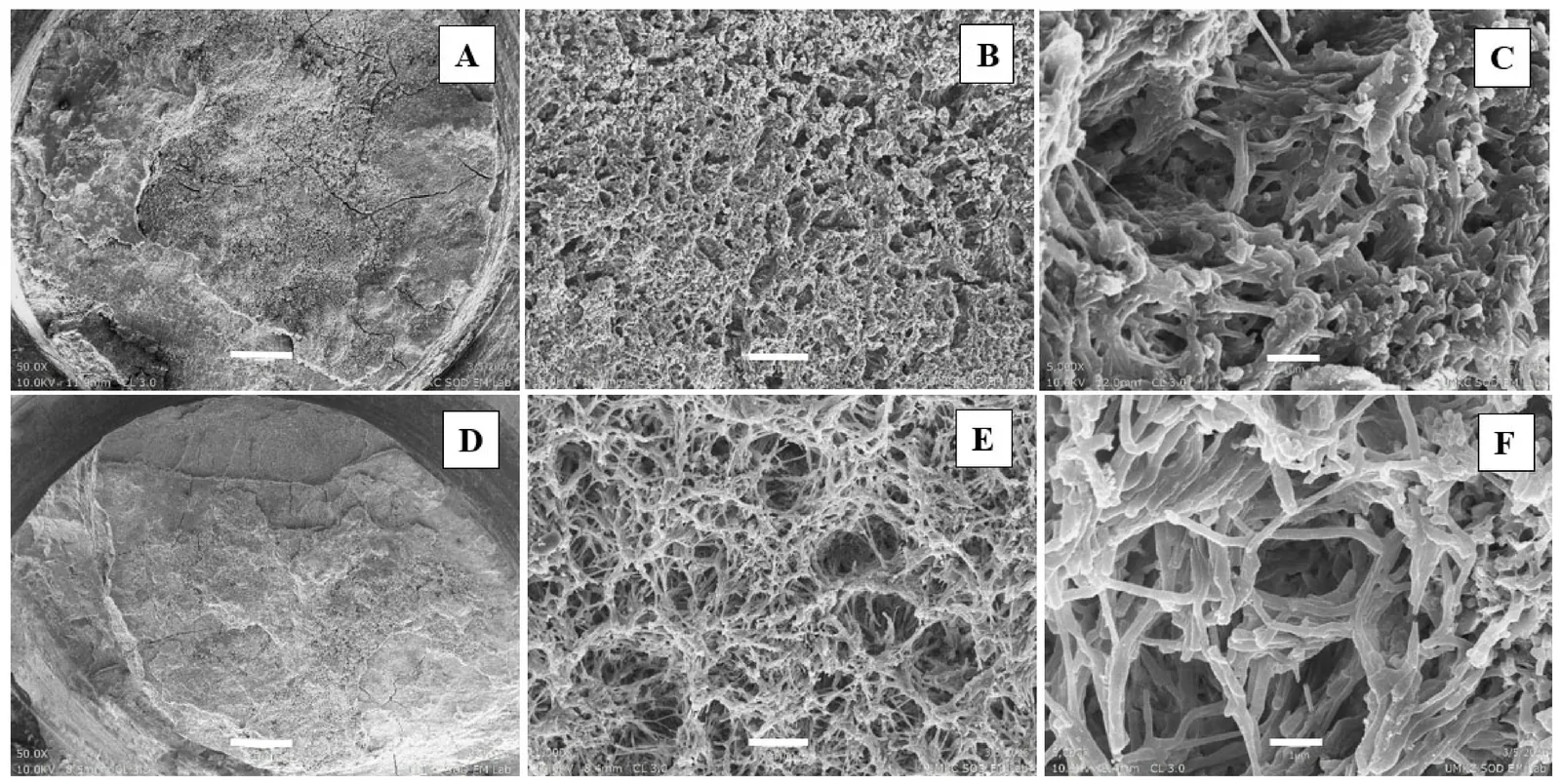
(**A**–**F**) Negative control (no treatment) and (**D**–**F**) positive control (air–water spray for 10 s). (**A**,**D**) Overviews of the circular inscribed treatment site (50×, bar = 300 µm). Both views show a rough surface of calculus and a surface layer of intact biofilm. The rough surface areas in (**B**,**E**) (1000×, bar = 30 µm) and (**C**,**F**) (5000×, bar = 3 µm) show undisturbed surface layers of biofilm; (**C**,**F**) shows, in detail, a biofilm mass consisting mostly of rod-shaped microbes of varying lengths and a scattering of cocci (**C**) and a biofilm mass consisting primarily of long rod-shaped microbes, similar in morphology to fusobacteria (**F**).

**Figure 3:**
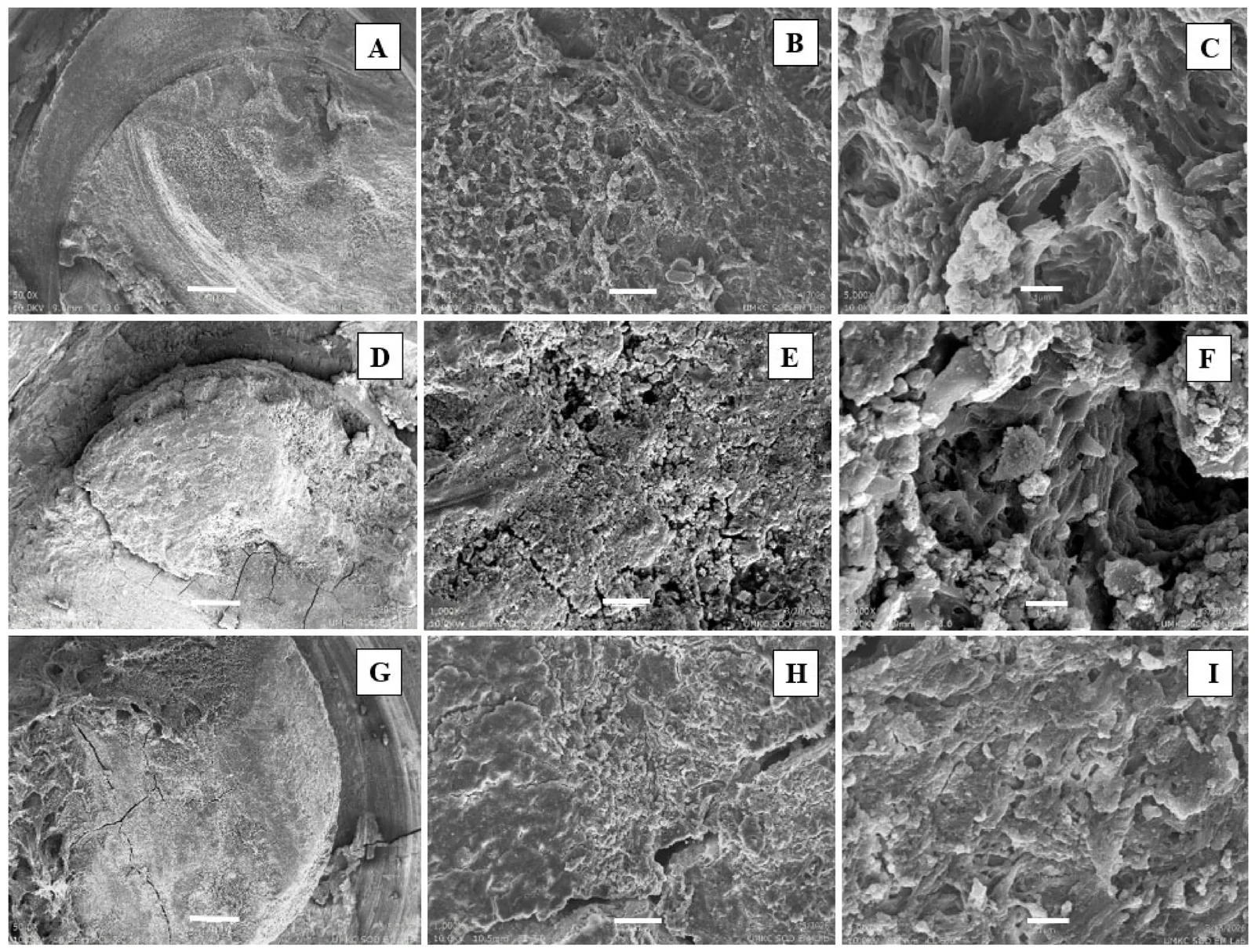
(**A**–**I**) Specimens from the air-polishing test groups: (**A**–**C**) represent a 5 s test specimen, (**D**–**F**) is a specimen from the 10 s test group, and (**G**–**I**) are from the 15 s test group. (**A**) (50×, bar = 300 µm) and (**B**) (1000×, bar = 30 µm) show residual calculus, a porous surface. (**B**) Porous surface consisting of ECPM devoid of surface microbes. (**C**) (5000×, bar = 3 µm) shows embedded rod-shaped microbes in the subsurface areas of residual calculus. (**D**) (50×, bar = 300 µm) shows residual calculus with both smooth and granular surfaces. At magnifications of 1000×, bar= 30 bar µm (**E**) and 5000×, bar = 3 µm (**F**), a rough granular surface and rod-shaped microbes embedded in ECPM are noted. (**G**) (50×, bar = 300 µm) and (**H**) (1000×, bar = 30 µm) reveal a distinct smear layer with adjacent areas of porous and granular surface texture (**H**). (**I**) (5000×, bar = 3 µm) reveals the smear layer to contain embedded rod-shaped microbes.

Table 1 presents the results of the SEM analysis of 21 specimens. Both negative (no treatment) and positive control (air–water spray) specimens exhibited residual calculus and a confluent layer of biofilm and ECPM covering most of the circumscribed areas of interest. The biofilm surface consisted of rod-shaped microbes of various lengths (Figure 2A–C and Figure 3A–C), the majority having a morphology consistent with fusobacteria (Figure 2C and Figure 3C). The similarity in negative and positive control group specimens suggests the air–water spray alone had minimal impact on the integrity and distribution of surface biofilm and associated ECPM.

As seen in Table 1, SEM images of specimens in each of the air polishing treatment groups yielded similar results. The 5 s air polishing treatment group (Figure 3A–C) showed a treatment effect that with longer exposure times appears to achieve minimal additional change, suggesting a time-independent effect. The initial treatment effect consisted of the removal of surface biofilm (Figure 3A,B), exposing a porous surface texture which at high magnification consisted of ECPM devoid of microbes (Figure 3B). However, it was also noted that subsurface areas of the ECPM contained embedded microbes, most of which exhibited a medium or long rod-shaped morphology (Figure 3C).

The 10 s treatment group exhibited residual calculus, with a more porous and somewhat granular surface with less obvious ECPM and no surface biofilm (Figure 3D–F). However, like the 5 s treatment group, the deeper areas of ECPM, when viewed at higher magnification, contained embedded microbes with rod-shaped morphology (Figure 3F).

The treatment effect in 15 s specimens (Figure 3G–I) was similar to other treatment groups with subtle differences. All three specimens exhibited localized areas of residual calculus, and two specimens exhibited isolated areas of a smear layer (Figure 3H) in which embedded microbes could be visualized (Figure 3I). One of the three specimens in the 15 s group exhibited a distinctly porous and granular surface texture in the treatment area. Two of the three specimens had smooth and rounded topographic features. One specimen exhibited an area of residual gingival fibers.

### 3.2. EDS Analysis

EDS data for the selected elements is displayed in Table 2, which represents an average of four EDS analyses taken from four areas on one specimen each of unused erythritol powder, untreated negative control, and 5, 10 and 15 s test samples.

**Figure 4:**
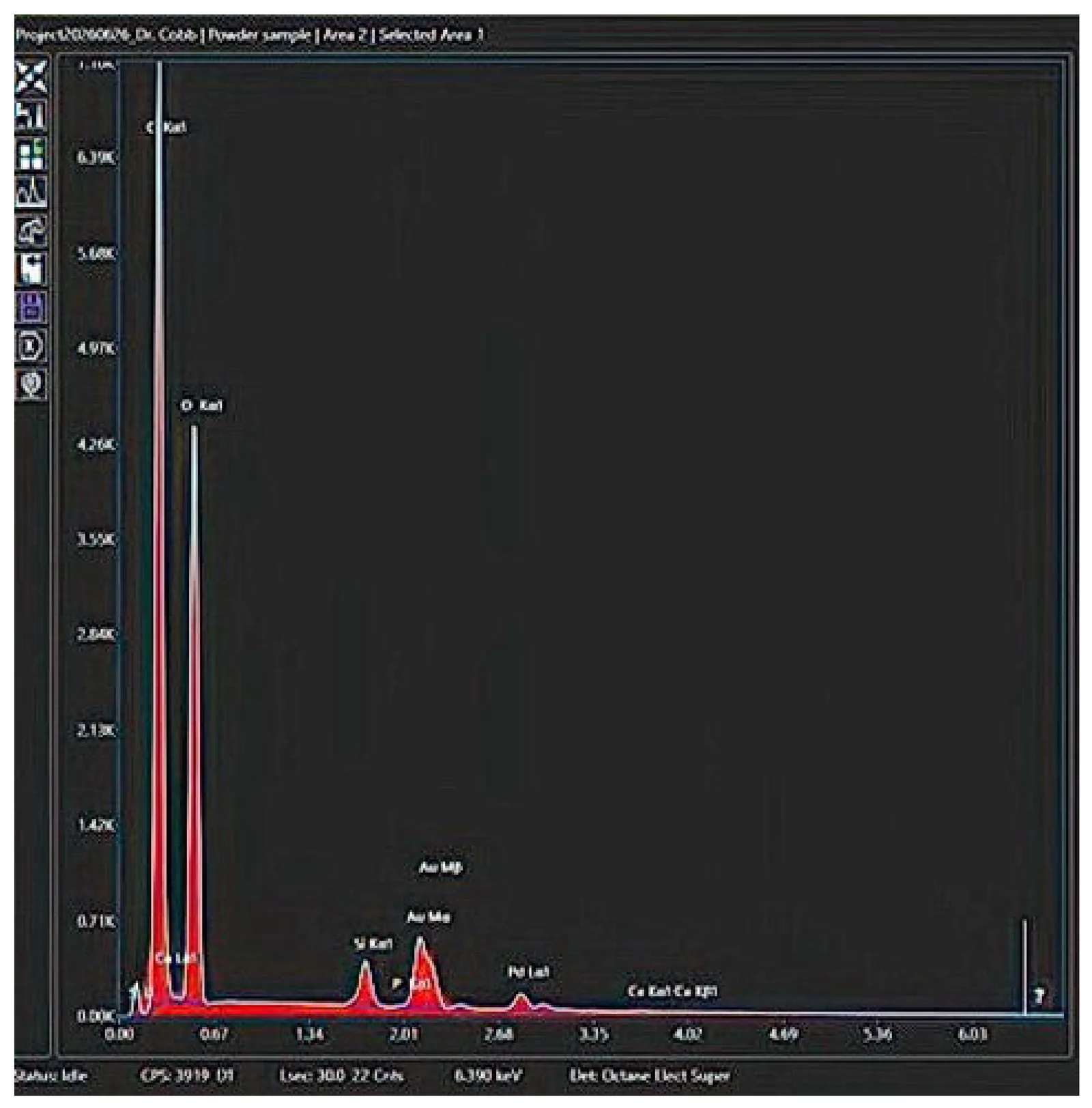
EDS scan of erythritol powder. From left to right, the peaks indicate the presence of carbon (C), oxygen (O), silicon (Si), gold (Au) and palladium (Pd). The gold and palladium are components of the sputter coating used to prepare specimens. The carbon, oxygen and silicon peaks are representative of the composition of commercial erythritol powder (C_4_H_10_O_4_). Silicon (2%) is added to commercial erythritol powder to achieve mild abrasive activity.

**Figure 5:**
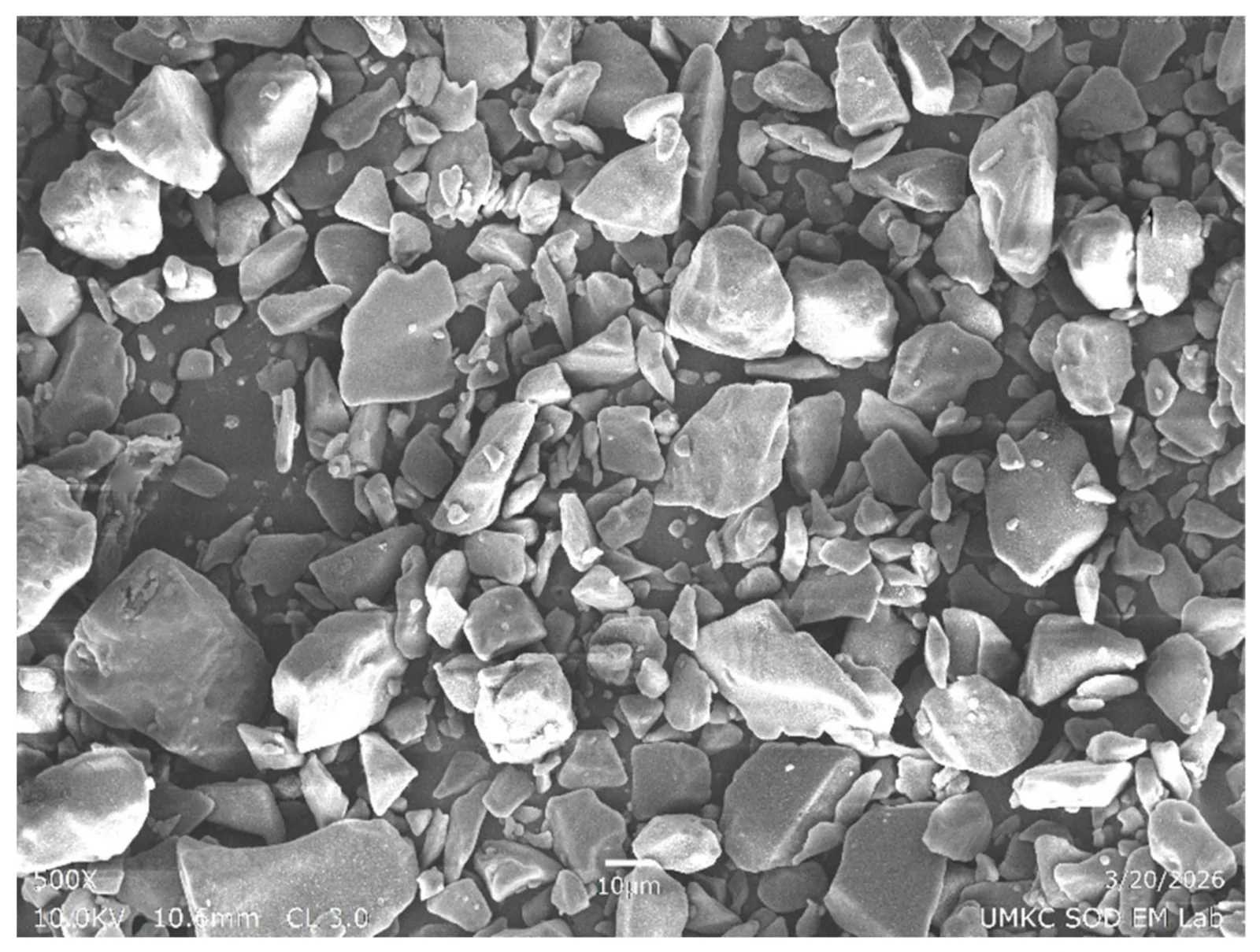
Low-magnification SEM view of the specimen of unused erythritol powder. Note the large variation in particle size comprising the erythritol powder sample in this view, ranging from ≈5 to ≈40 µm. Original magnification of 500×. Bar = 10 µm.

The chemical composition of commercial-grade erythritol powder (C_4_H_10_O_4_) with silicon added to achieve a low-level ablativity can be seen in the weight % and atomic % data, respectively, i.e., carbon (C) at 66.2% and 73.9%, oxygen (O) at 25.6% and 21.8%, and silicon (Si) at 2.6% and 1.1%. Figure 4 shows the corresponding peaks on the EDS scan. The SEM image used to delineate the EDS scan areas shows a large variation in the particle size of the erythritol powder sample, ranging from 7.5 to 37.5 µm (Figure 5).

The EDS data from the negative control specimen compared to that of the test specimens showed a decreases in weight % for calcium (Ca), phosphorus (P) oxygen (O), and sulphur (S), an increase in carbon (C), and essentially no change in sodium (Na), magnesium (Mg), or silicon (Si).

## 4. Discussion

Recently, the proponents of air polishing have advocated a protocol referred to as Guided Biofilm Removal (GBR) or Guided Biofilm Therapy (GBT) [18,19,20]. The protocol is promoted as a new and novel approach in dental biofilm management [18,19,20,21,22,23] and is reported to be beneficial in the removal of “young” calculus and biofilm around teeth and dental implants, resulting in a reduction in microbial load and inflammatory cytokines [18,19,20]. The GBR/GBT protocol uses a disclosing agent for the visualization of the biofilm, and the removal of the biofilm is performed with an air polishing unit using a fine-particle-sized erythritol powder, with removal of calculus using ultrasonics and/or manual instrumentation [23]. The protocol is advocated to be of benefit in periodontal pockets with probing depths ranging from > 4 to 9 mm [5].

The belief that air polishing with erythritol powder removes “young” calculus and biofilm in periodontal pockets up to 9 mm should be accepted with caution. First, as previously noted, “young” calculus is imprecisely defined. Different crystal forms of calcium phosphate can be present in calculus, including hydroxyapatite, magnesium whitlockite, octacalcium phosphate, and brushite [12]. The mineral content of immature calculus is primarily brushite, whereas that of subgingival mature calculus consists of brushite, whitlockite, hydroxyapatite and its precursor octacalcium phosphate [6,13,24,25,26]. Subgingival dental calculus tends to have a higher ratio of calcium to phosphorus compared to that of supragingival calculus [27]. Supragingival immature calculus is heterogeneous, with islets of mineralized material and nonmineralized areas within the calculus [7,28]. In contrast, subgingival mature calculus is more homogeneously calcified [7,28]. Both types, immature and mature, exhibit a surface layer of unmineralized bacterial biofilm and, depending on the thickness of the deposit, can have pockets of viable anaerobic bacteria within the calculus mass [29,30,31,32]. This latter observation was confirmed in the present study with SEM evidence of rod-shaped microbes embedded in residual ECPM in all specimens, regardless of treatment group. Obviously, residual calculus with embedded viable bacteria can be responsible for relapsed treatment and/or progression of disease [29,30,31,32].

Secondly, the GBR/GBT protocol stipulates the removal of subgingival calculus after air polishing for the removal of biofilms, both supra- and subgingival. The question arises, in periodontal probing depths of ≥ 4 mm, of whether the GBR/GBT protocol produces results that are superior to those achieved by traditional scaling and root planning (SRP) using ultrasonic and/or manual instrumentation. It is well established that as probing depth increases there is a concomitant decrease in the efficacy of dental calculus removal using traditional SRP techniques [33,34,35,36,37]. Since air polishing alone does not remove mature subgingival calculus [1], such removal must rely on traditional instrumentation. Thus, for the elimination of subgingival calculus at deeper probing depths, the GBR/GBT protocol is confronted with the same challenges as traditional instrumentation protocols. In that regard, it is interesting to note that recent clinical studies comparing GBR/GBT to conventional non-surgical therapy in the initial treatment phase of stages III and IV periodontitis patients reported comparable results in reductions in periodontal probing depth, bleeding on probing, pocket closure, and microbiologic outcomes [20,38,39,40,41,42,43,44].

In the current study, the presence of residual calculus was observed in all (9 of 9) air-polish-treated specimens, with time of treatment having no effect. The lack of effect with increasing time of treatment is likely related to the degree of mineralization of the residual calculus (mature vs. “young” calculus), the thickness of the polymeric extracellular matrix, and the depth of embedment of residual bacteria in mature calculus.

The presence of a smear layer was noted in five of the nine air-polish-treated specimens, with embedded microbes observed in all five specimens. Lastly, microbes embedded in residual calculus were observed six of nine air-polish-treated specimens. This latter observation is clinically relevant as smear layers have been described as containing not only bacteria but also endotoxins and contaminated cementum [45,46,47]. If these findings reflect the in situ condition, then definitive non-surgical treatment must still rely on SRP for the removal of residual biofilm, contaminated calculus, and contaminated smear layers.

Regarding reduction in subgingival biofilm, studies by Moene et al. [3] and Wennström et al. [4] came to the same conclusions in their respective clinical trials, comparing SRP alone to air polishing with glycine powder in the treatment of periodontitis, i.e., that the air polishing technique offers more patient comfort vs. traditional therapy but that there was no statistically significant difference in microbiological variables between treatment modalities.

An evidence-based consensus statement, published in 2024 [48], reported that air polishing with both glycine and erythritol was efficient, safe, and comfortable for the patient. However, the consensus report stated that more evidence was required to support claims of improvement in clinical attachment level, especially when erythritol was used. Additionally, the report noted that more long-term evaluations were needed on the effects of subgingival air polishing on subgingival biofilms and the inflammatory response, particularly in light of the GBR/GBT protocol (“concept”).

Somewhat in contrast is a study by Divnic-Resnik et al. [22] that compared subgingival air polishing therapy using erythritol powder to conventional debridement with ultrasonic and manual instrumentation in stage II and III periodontitis patients. At 6 months post-treatment, the authors reported that air polishing therapy resulted in a significantly greater number of initially deep pockets (≥5.5 mm) being reduced to shallow pockets (≤3.4 mm). Concomitantly, the study showed a greater attachment gain in air-polish-treated sites. Interestingly, there were no reported microbiological differences between treatment groups. With respect to the latter observation, Mensi et al. [38] reported that GBR/GBT using erythritol-chlorhexidine powder, compared to conventional treatment (ultrasonic SRP), appeared to cause a shift in the periodontal microbiome towards a more eubiotic state. However, other studies reporting on the microbiological impact of subgingival air polishing using erythritol powder noted no differences when compared to traditional SRP [43,49,50,51,52].

Subgingival air polishing may offer potential benefits for patient comfort and shorter treatment times [43,52]. However, recent systematic reviews and meta-analyses report that air polishing and traditional SRP are both clinically effective in disrupting biofilm, reducing periodontal inflammation, and improving key clinical parameters [43,51,53,54]. Indeed, if patient-reported outcome measures are excluded (e.g., discomfort and time of treatment), air polishing using erythritol or glycine protocols do not show superior clinical outcomes versus traditional SRP.

Given the conflicting results when comparing erythritol air polishing to more traditional treatment modalities as regards reduction in the subgingival microbiome, it appears that the major benefits derived from the GBR/GBTR protocol are patient comfort and ease of application, with no significant differences in clinical outcomes. Indeed, a critical evaluation of the GBR/GBT protocol reveals that the only “new” part of the concept is the use of the air polishing instrumentation with the fine-particle-size powder. All other steps in the protocol are typically employed in traditional treatment, which likely accounts for the similarity in clinical outcomes regardless of treatment protocol.

EDS weight % and atomic % indicate the relative concentration of the element in the sample, with weight % signifying the portion of the total sample mass contributed by a specific element and the atomic % being a direct measure of atomic abundance of individual elements in the sample. The manufacturer of the erythritol powder used in this study states that there is “less than” 2% amorphous silicon, a statement confirmed in this study by the EDS results of the unused powder sample (weight % of 2.3). Interestingly, levels of silicon never reach zero in the test specimens (weight % range of 0.2 to 0.8). This finding may have clinical significance in that residual silicon has been associated with gingival white verrucous lesions. Indeed, silicon was reported to be the most common foreign material in such lesions [55].

Ex vivo experimental models have attempted to create an environment that simulates the in situ milieu. However, given the inherent nature of ex vivo studies, they cannot fully achieve duplication of the clinical environment. Despite the inherent problems with such a system, ex vivo experimental models are valuable for proof of principle studies. With that consideration, the results of the current study may have been impacted by several issues: (1) air polishing may be technique-sensitive and therefore produce a variable effect; (2) criteria used for evaluating specimens may have lacked powers of discrimination; (3) effects of air polishing were too subtle to differentiate using SEM; and (4) the enclosed area of a pocket, unlike the open ex vivo environment, may facilitate the removal of biofilm through the creation of a pressure irrigation effect. Accurate determination of the effects of air polishing on calculus and biofilm in 4–9 mm periodontal pockets will require in situ treatment of diseased teeth planned for removal and subsequent examination using discriminatory magnification levels of SEM.

## 5. Conclusions

The results of this ex vivo pilot study indicate that air polishing using erythritol powder of calculus-ladened root surfaces for 5, 10 and 15 s did not remove subgingival calculus or embedded microbes. Further, following air polishing treatment, a smear layer was noted on roughly one half of treated specimens, with microbes embedded in the smear layer. Thus, the clinical application of erythritol air polishing protocols must still rely on subgingival scaling and root planning to achieve the desired endpoints of periodontal therapy.

## Figures and Tables

**Table 1:** Evaluation of surface characteristics of the five specimen groups. Number and percentage of specimens exhibiting the listed surface feature.

Surface Characteristics	Negative Control (No Treatment) n = 3	Positive Control (Air–Water Spray) n = 8	5 s Treatment n = 3	10 s Treatment n = 3	15 s Treatment n = 3
Presence of calculus	3 (100%)	7 (88%)	3 (100%)	3 (100%)	3 (100%)
Presence of smear layer	0	0	1 (33%)	2 (66%)	2 (66%)
Instrumentation marks	0	0	1 (33%)	2 (66%)	2 (66%)
Granular debris field on surface	0	2 (25%)	1 (33%)	1 (33%)	1 (33%)
Smooth, flat or rounded surface topography	0	4 (50%)	1 (33%)	1 (33%)	2 (66%)
Rough surface topography	3 (100%)	8 (100%)	2 (66%)	2 (66%)	1 (33%)
Porous surface texture	0	0	1 (33%)	1 (33%)	1 (33%)
Presence of biofilm	3 (100%)	7 (88%)	1 (33%)	1 (33%)	1 (33%)
Bacteria embedded in calculus	3 (100%)	8 (100%)	2 (66%)	2 (66%)	2 (66%)
Evidence of an ECPM	3 (100%)	8 (100%)	2 (66%)	2 (66%)	2 (66%)

**Table 2:** EDS semiquantitative data for selected elements. Data represents an average of scans taken from four areas on one specimen each of unused erythritol powder, untreated negative control, and 5, 10 and 15 s test samples.

Element	Erythritol Powder		Negative Control		5 s Test		10 s Test		15 s Test	
	Weight %	Atomic %	Weight %	Atomic %	Weight %	Atomic %	Weight %	Atomic %	Weight %	Atomic %
Ca	0.3	0.1	30.8	16.0	15.0	6.1	17.8	8.3	22.9	10.4
P	1.9	0.4	17.6	11.8	10.8	5.7	13.5	8.0	10.3	6.2
C	66.2	73.9	12.5	21.6	41.8	55.4	26.5	37.1	31.5	48.4
N	1.0	1.0	4.1	6.0	11.6	13.2	8.6	11.6	5.3	7.0
O	25.6	21.8	33.5	43.5	19.0	16.0	29.8	33.9	23.7	27.7
Na	0.0	0.0	0.2	0.2	0.5	1.1	0.4	0.3	0	0
Mg	0.1	0.1	0.1	0.1	0.4	0.9	0.9	0.7	0	0
S	2.6	1.1	1.2	0.8	1.6	1.2	1.2	0.6	2.2	1.3
Si	2.3	1.1	0.3	0.2	0.3	0.2	0.8	0.5	0.2	0.1

## Data Availability

The raw data supporting the conclusions of this article will be made available by the authors on request.
